# Measuring the “Sandwich”: Care for Children and Adults in the American Time Use Survey 2003–2012

**DOI:** 10.1007/s10834-016-9483-6

**Published:** 2016-02-20

**Authors:** Jooyeoun Suh

**Affiliations:** Center for Time Use Research, University of Oxford, 74 Woodstock Road, Oxford, OX2 6HP UK

**Keywords:** Child care, Adult care, ADLs and IADLs, Sandwich caregivers, American time use survey

## Abstract

While the economic burden of simultaneously caring for young and old family members is widely recognized, it has yet to be accurately measured. Yet, such assessments are relevant both to public policies providing support to family caregivers and to private insurance markets for long-term care. This descriptive study presents a new method to address this problem: the construction of a crosswalk between time-use diaries and other types of surveys using lists of activities of daily living (ADLs) and instrumental activities of daily living (IADLs) for which assistance is required. Analysis of pooled data from American time use survey 2003–2012 provides some quantitative indicators, but understates the temporal burden of care and fails to distinguish types of care that involve personal interaction from those that do not. A crosswalk of time-use survey categories with the list-based approach typically applied in public health surveys clearly demonstrates the importance of clear definitions and also offers more precise measures. Depending on how sandwich caregiving was defined, the temporal burden for caregiving ranged from 11.2 to 60 h per week, clustering at around 20 h per week for most cases. This result demonstrates the magnitude of sandwich care demands and also underscores the need for improved care survey design. As shown in this study, such efforts should take into account the implications of disaggregating data by gender and age, and definitional variations that characterize existing datasets.

## Introduction

As age at first birth has increased, along with life expectancy, the probability that adults will face responsibilities for care of both young children and elderly parents has increased. Nearly half (47 %) of Americans aged between 47 and 59 have an older parent aged at least 65 years and are also raising at least one child aged under 18 or provide financial support to a grown adult child aged 18 and over (Parker and Pattern 2013). So-called “sandwich” caregivers tend to care for both young and old family members in need of assistance. The term can also be applied to those caring for both children and for adults who are suffering from illness or disability, regardless of age. Time-use surveys such as the American time use survey (ATUS) have considerable potential to help measure the temporal burden of sandwich care. Unfortunately, this potential has been limited by conceptual inconsistencies, which have led to serious measurement problems. As a result, it is difficult to accurately assess the amount of time devoted to sandwich care on a given day or to calibrate estimates of daily care demands with estimates of the frequency of assistance provided over a longer time period.

This paper explores these measurement problems, showing that they reflect failure to clearly conceptualize the temporal burden of care and to distinguish types of care that involve personal interaction from those that do not. Next, it develops a strategy for working around these problems, offering upper- and lower-bound estimates of average time devoted to sandwich care in the US based on the ATUS. These alternative estimates have important implications for assessing the relative burden of care for children and adults and the contributions of women and men. Many of the insights that emerge from this empirical exercise are relevant to surveys regarding assistance provided to adults with activities of daily living (ADLs) and Instrumental Activities of Daily Living (IADLs). The construction of a crosswalk between time-use and other types of surveys offers a promising strategy for improved measurement. The paper concludes with an emphasis on the need for improved survey design.

## The Temporal Burden of Care

Care for family members in need of assistance is time-consuming and potentially costly, often leading to rearrangement of employment schedules, unpaid leaves, or even exit from the labor force (Bianchi et al. 2006; Wolf and Soldo 1994; Zuba and Schneider 2013). Women have taken on disproportionate responsibility for child care and adult care regardless of their employment status (Bianchi 2000; Moen et al. 1994; Nichols and Junk 1997). One study showed that approximately 66 % of family caregivers are women (National Alliance for Caregiving in collaboration with AARP 2009). Another study focusing on the characteristics of elder caregivers, by Stone et al. (1987), based on the 1982 National long-term care survey (NLTCS), showed that a majority (71.5 %) is female. Mothers have been far more likely than fathers to withdraw from the labor force or reduce their hours of work to provide care to a family member (Craig 2006; Molina 2015; Reynolds and Aletraris 2007).

The relative number of individuals who are both raising children and caring for parents has grown as baby boomers have advanced toward the threshold of old age. The fertility rate for baby boomers has been about or less than 2 children, compared with rates of between 2.4 and 3.6 children for their parents’ cohorts (Spillman and Pezzin 2000). Additionally, as life expectancy increases, more middle-aged people have living parents. The size of the sandwich generation depends on how one defines it. Henretta et al. (2001) have defined the sandwich generation as women ages 55–63 (born between 1931 and 1941) living with both children and her parents. Between 32 and 37 % of women in this age group have both living children and at least one living parent, with highly educated women (more than 12 years of education) more likely than women with less education (<12 years of education) meeting this criterion (Henretta et al. 2001).

The availability of help or support from other household or non-household members also has had an impact on primary caregivers’ time allocation. Using 1999 NLTCS data, Rubin and White-Means (2009) found that sandwich caregivers were more likely to get help from secondary caregivers compared to non-sandwiched caregivers. Although the total numbers of hours of caregiving would be higher for sandwich caregivers, if the secondary caregiver is available, the caregivers were more likely to spend less time on caregiving for the elderly (Rubin and White-Means 2009).

Care demands are shaped by the age of care recipients. The temporal demands of child care are relatively predictable, and typically decline as children age. The temporal demands of adult care are less predictable.^1^ The aging process affects individuals quite differently, often leading to episodic health problems, but also involving chronic conditions that tend to worsen over time. But age is not the only factor determining care needs. About half the adults in the US needing assistance with performing daily activities (self-care activities, such as bathing and dressing, and other routine activities, such as shopping and doing housework) who are living outside institutions have been found to be non-elderly (Kaye et al. 2010).

### Conceptual and Measurement Issues

While many time-use researchers have sought to measure the temporal demands of care for children and adults needing assistance due to aging or disability, they have also acknowledged serious measurement problems (Bianchi et al. 2006; Folbre et al. 2005; Folbre and Wolf 2013). Like many other time-use surveys, the ATUS has failed to distinguish between time spent caring for elderly adults and those with non-age-related disabilities (Budlender 2008).^2^ Further, time-use surveys typically have sampled only 1 day in the year, yet the demands of adult care are often distributed very unevenly throughout periods longer than a day, often much more so. As a result, individuals currently providing care to adults almost certainly have been under-sampled relative to adults currently providing care for children.

More serious measurement problems derive from conceptual ambiguities. Much of the time-use literature focusing on care has emphasized the distinctions among interactive care activities involving personal and often emotional contact, supervisory care or “on-call” responsibilities, and support care activities such as housework that develop and maintain an environment for interactive care (Albelda et al. 2009; Allard et al. 2007; Bianchi et al. 2006; Folbre 2012). Even though supervisory and support care tasks may be performed outside the immediate presence of a care recipient, they are often customized to that recipient’s special needs. In many ways the distinction between interactive care and support care echoes the distinction between two categories used in surveying episodes of care. The category of “Assistance with Activities of Daily Living” (ADLs) entails help with essentially personal activities such as eating, using the bathroom, and getting dressed. The category of “Assistance with Instrumental Activities of Daily Living” (IADLs) entails less personal activities such as shopping and paying bills (Levine et al. 2003). Table 1 lists a standard designation of activities.

**Table 1:** Standard lists for activities requiring assistance

Activities of daily living (ADLs)	Instrumental activities of daily living (IADLs)
Bathing or showering	Light housework, heavy housework, or work around the house or yard
Dressing	Doing laundry
Eating	Preparing meals
Getting in and out of bed	Shopping for groceries or personal items
Using the toilet	Making phone calls or using telephone
Getting around inside or walking across a room or walking	Taking or managing medication
	Managing money

Measures of ADLs and IADLs vary by surveys. However, I take the common ADL and IADL activities in following surveys: health and retirement study (HRS), National Health Interview Survey (NHIS), National Long-Term Care Survey (NLTCS), and Survey of Income and Program Participation (SIPP)

Lack of attention to these conceptual nuances has often led to operational differences in the definition of time devoted to care. Most empirical research has focused on interactive care activities, which are defined fairly consistently for the care of children and adults. Some small anomalies, however, are apparent even in this category. For instance, activities related to education such as homework and home schooling are more relevant to children than adults. Leisure-related activities also differ. “Playing with children” is considered a form of child care, but there are no categories of “playing” with adults. The boundary between leisure and adult care is difficult to identify. Engaging in social interaction with adults who would otherwise feel isolated probably represents an important aspect of emotional care, but has not been coded as such in the ATUS. Some surveys of elder care in particular, such as the National Health and Nutrition Examination Survey (NHANES), have explicitly asked respondents about difficulty with attending movies or sporting events; participating in social activities (visiting friends or going to parties); and doing things to relax at home or for leisure (reading, watching TV, sewing, listening to music).

#### Issues Related to Supervisory Care

More serious problems concern the treatment of supervisory care, especially in combination with distinctions between care for household and non-household members. Supervisory care, sometimes termed “on call” time, has described a responsibility rather than an activity, but may seriously constrain a caregivers’ ability to engage in paid employment or other productive activities (Bianchi et al. 2006; Budig and Folbre 2004; Folbre 2012). Researchers now widely acknowledge the significance of supervisory care of children, but continue often to overlook supervisory care of adults, although this can be extremely demanding for family members with severe mental or physical disabilities (Folbre 2012; Folbre and Yoon 2007; Moore et al. 2001).

The ATUS has asked respondents to report the amount of time that a child under the age of 13 was “in your care,” tabulating this as “secondary child care.”^3^ There has been no corresponding question for adult care.^4^ Yet long-term care researchers focusing on the elderly and individuals with disabilities have observed that “supervisory help” is both time-consuming and likely to be under-reported by family members (Levine 2004, 2012). One effort to address this problem is a Caregiver Vigilance Scale that asks caregivers to assess subjective and temporal burdens in addition to ADL assistance and IADL assistance. The Caregiver Vigilance Scale has been widely applied in health-related research, because of its relevance to treatment of those suffering from Alzheimer’s disease or related disorders (Carr 1997; Gitlin et al. 2003; Mahoney et al. 2003).

Supervisory care for adults has not, however, received much attention in the social science or time-use literature, and this creates difficulties for calculation of sandwich care demands. In principle, supervisory care for children should be included in a measure of temporal burden. However, lack of a parallel category for supervisory care of adults who are suffering from illnesses, disabilities, or simple frailties of old age has understated the burden of care for adults relative to care for children when this larger definition is applied.

#### Issues Related to Support Care

Support care is another dimension that has often been inadequately considered when dealing with children and frail adults. Support care activities are those that may not involve direct interaction but set the stage, in a sense, for interactive care. Time-use researchers have seldom included support care in measures of total time spent on children because there are no questions asking “who for” in time-use surveys. Stylized surveys^5^ of care activities, on the other hand, have taken a more inclusive approach, often ignoring the distinction between interactive and support care. Asked how many times in the previous month or year they provided “care” for an elderly parent, most respondents would probably include both types of activities—preparing meals or running errands as well as feeding or bathing.

In a sense, housework and household organization represent public goods that benefit all household members. When an adult lives in a separate household, however, it is easier to identify the specific beneficiary of assistance with these activities. Perhaps for this reason, the ATUS has explicitly measured support care for adults living in separate households—such as preparing their meals, doing their laundry, or mowing their lawn. The same activities conducted on behalf of a resident adult, however, have not been explicitly measured. Two coding categories, caring for household adults (or non-household adults) and helping household adults (or non-household adults), clearly illustrate this problem. The list of sample activities for helping household adults has implied that such adults are dependent, while the list of activities for non-household adults has included a much longer list of activities that encompasses housework and related activities. In other words, it appears that “support care” such as housework or help with instrumental activities of daily living (IADLs) has not been separately tallied for household adults, but has been separately tallied for non-household adults. This inconsistency could significantly bias comparisons of these two categories of care. Surveys of frequency of assistance show that most of the help with IADLs have come from non-household rather than household caregivers. For instance, non-household caregivers have provided more of the help with “getting around outside,” travel beyond walking distance, and financial management tasks (Wolf 2001).

These apparently minor differences in activity code can have significant implications for the measurement of the temporal burden of sandwich care. Men may have been more likely to provide support care for non-household adults, such as running errands or doing yard work. Women may have been even more likely to provide support care for household adults that is not distinguished from the larger category of household support work (Folbre 2012). If the category “Helping Non-Household Adults” is included in a measure of adult care, this definitional inconsistency makes it appear that men have provided a greater percentage of elder care than would be the case if support care were treated consistently whether provided in one’s own or another household.

#### Inconsistencies Between ATUS and List-Based Measures

A final measurement issue concerns lack of consistency between the activities and responsibilities coded in the ATUS and surveys applying list-based measures such as ADLs and IADLs. Many US surveys, including the health and retirement study (HRS), longitudinal study of aging (LSOA), and the National long-term care survey (NLTCS), have asked respondents to report the number of episodes in which they provided help to a family member or other person (i.e., care episodes). These categorical lists have also been widely applied in assessments of need for institutional assistance, such as nursing home care. Yet these lists have not included any explicit consideration of the amount of time devoted to specific forms of assistance.

Surveys of elder care in particular focusing on ADLs and IADLs have been extremely varied in their wording. For instance, some surveys have asked about ADLs or IADLs separately, while others have grouped them together into just one or two global questions. The Health and Retirement Survey (HRS) has asked separately about the basic six ADL activities (dressing, walking across a room, bathing or showering, eating, getting in or out of bed, and using the toilet, including getting up and down). On the other hand, the National Health Interview Survey (NHIS) has asked only a single question about ADLs. The activity-by-activity approach used in the HRS has given caregiver respondents more chances to report their caregiving burdens, and might therefore have generated more reports than the global approach taken by the NHIS. Efforts to crosswalk and calibrate these different measures have had limited success (Freedman et al. 2004; Wiener et al. 1990).

Time-use surveys have the potential to complement surveys of care that capture numbers of episodes, as in HRS, LSOA, and NLTCS, and vice versa. While the ADL-IADL measures have been sufficient for some caregiving assessments, they have not addressed the scope and complexity of many caregivers’ responsibilities. These have included medical tasks, coordination with care recipients, and management tasks, which are activities often overlooked by long-term-care researchers (Levine 2004). Also, caregivers do not always think of what they do in terms of ADLs and IADLs. Rather, they do whatever needs to be done. As a result, a list-based measure can lead to underestimates of the total work that they perform.

In sum, efforts to measure the temporal burden of care for children and adults needing assistance have suffered from a variety of methodological limitations. Definitions of what constitutes unpaid care have varied between child care and adult care even within the same survey; variation across different types of surveys has also been problematic. Nonetheless, careful analyses of the ATUS, sensitive to the problems outlined above, offer some insights into the relative burdens of child care and adult care and how these are distributed between men and women.

### Research Questions

My analysis of the temporal burden of sandwich caregiving based on the ATUS asks four questions: (a) what are the implications of different definitions of care for the assessment of the relative share of adults engaged in sandwich care on a given day? (b) given the definitional ambiguities, what are reasonable lower- and upper-bound estimates of the relative share of adults engaged in sandwich care and the average amount of time they devote to such care? (c) what are the implications of these estimates for consideration of the relative burden imposed by child care and adult care, and the relative burden on women and men caregivers? and (d) how do these estimates of the amount of time devoted to care compare with estimates based on stylized surveys inquiring about episodes of assistance with ADLs and IADLs?

## Data and Methods

The American Time Use Survey 2003–2012 (ATUS) is a nationally representative survey that collects information on how non-institutionalized individuals in the United States aged 15 and over spend their time during a representative day (the day of survey). The information on how individuals use their time is collected in phone interviews during which respondents sequentially describe each of their main activities, along with the duration of activity, and start and end times. Each of these activities is subsequently coded into one of over 400 detailed activity categories. Interviews are conducted every day except for few major holidays like Thanksgiving Day and Christmas Day.

This paper focuses on sandwich caregivers, individuals aged 18 and over who spent some time on both child care and adult care during survey day. The estimates were reported across gender and age groups (18–24, 25–44, 45–64, 65 and over). Analysis samples included 3669 female caregivers and 1,819 male caregivers in data pooled over the 2003–2012 period. As emphasized above, definitions of both child care and adult care vary, both within the ATUS and between the ATUS and stylized surveys of care episodes. Hence, a range of estimates based on different definitions, including a lower-bound and an upper-bound, provides a more reliable picture than a single estimate. The ATUS sample weights were used throughout the analysis. Weighting was necessary to correct for the stratification of the sample and for differential response rates across groups.^6^

This analysis classified child care into two categories: interactive child care and supervisory child care. Interactive child care included four different types of activities: physical care (feeding, bathing, etc.), developmental care (talking to or reading aloud to children), managerial care, and traveling associated with interactive child care activities (including waiting for children at the doctors’ office). Supervisory care was defined by the amount of time reported in response to the question, “whether your child was in your care?”, distinguishing it from interactive child care activities. Only supervisory care non-overlapped with interactive child care was considered in order to avoid double counting. (A detailed list of ATUS categories in child care and adult care can be obtained from the author.)

Interactive care for adults consisted of three activities: those activities coded as caring, helping, and traveling related to caring and helping. As discussed previously, “helping” activities for adults are treated differently for household adults and non-household adults, unlike interactive activities for children, which list the same activities for both household children and non-household children. While activities listed under “helping” for household adults include organizing or planning for adults, “helping” for non-household adults includes housework, cleaning, cooking, and so on, similar to support care. As discussed earlier, ATUS does not, unfortunately, collect data on supervisory care for adults needing assistance.

Included in the analysis is support care, such as housework, that helps create and maintain an environment for interactive care, along with other types of care, for individuals who engage in interactive or support care. In the absence of a question asking “for whom” an activity was conducted, there was no way of identifying the specific beneficiary of support care. Indeed, people living alone devote substantial time to “support care” for themselves, such as cooking and cleaning. In estimating support care, I assumed that this has substantial public good characteristics because, for example, laundry or dishes are done by mostly one person (usually women) in the household for all household members. In order to get a per capita measure, the total amount of support care was divided by the number of household members, as if support care were carried out individually. This per capita measure was then subtracted from the total amount of support care to exclude support care that could be construed as personally benefiting the caregiver.

In order to provide comparability with stylized surveys based on ADLs and IADLs, I broke out detailed activity descriptions from the ATUS that resemble these. As aforementioned, the distinction between ADLs and IADLs resembles the distinction between interactive care and support care that is often made in the time-use literature. However, ADLs and IADLs have traditionally been applied only in the gerontology literature, and are seldom, if ever, applied to child care.

While the match between ADLs and IADLs and ATUS categories is an approximate one, it provided at least some comparability across different types of surveys (details of the cross walk can be obtained from the author). The exercise revealed that family caregiving involves complex activities embedded in but not conventionally captured by ADL/IADL measures. For instance, the time devoted to travel as an IADL category was accurately measured in ATUS, whereas other categories of IADLs were only loosely matched with ATUS activities. Beyond ADL/IADL measures, caregivers monitored and supervised the care recipient’s behaviors, managed and organized paid care services, managed medical equipment, provided skilled nursing care, and so on. Especially for child care, care time devoted to children’s education for development, which is omitted in interactive adult care activities, was included.

## Results

### Participation in Care for Children and Adults

The analysis began by examining measures of participation in unpaid care for children and adults generated by pooling data from 2003 to 2012 ATUS. Table 2 shows the percentage engaging in caregiving by gender and age group for seven different definitions of caregiving (from non-sandwich caregivers to the most expansive definition of the sandwich caregivers). Panel 1 focuses on interactive child care, revealing gender and age differences similar to those reported elsewhere. Women ages 25–44 years made up the prime child care age group, and 62.6 % of those engaged in interactive child care activities, whereas only 7 % of women aged 65 and over engaged in interactive child care. For men, those in the prime age for child care (25–44) engaged in child care the most (40.5 %), while men aged 65 and over engaged in it the least (5.2 %). In every age category, women were more likely to provide child care than men.

**Table 2:** Participation on interactive child care and adult care, by gender and age of unpaid caregivers (ATUS 2003–2012)

	Interactive child care		Interactive adult care		Total interactive care (for children and adults)	
	Women	Men	Women	Men	Women	Men
	Engaged in activity on diary day	Engaged in activity on diary day	Engaged in activity on diary day	Engaged in activity on diary day	Engaged in activity on diary day	Engaged in activity on diary day
Panel 1: individuals 18 and over who engaged in any interactive child care						
Age of caregivers						
18–24	35.7 %	11.8 %				
25–44	62.6 %	40.5 %				
45–64	23.5 %	17.3 %				
65 and over	7.0 %	5.2 %				
Panel 2: individuals 18 and over who engaged in any interactive adult care						
Age of caregivers						
18–24			16.2 %	18.5 %		
25–44			12.2 %	11.6 %		
45–64			15.2 %	12.3 %		
65 and over			11.6 %	13.2 %		
Panel 3: individuals 18 and over who engaged in any interactive child care or interactive adult care						
Age of caregivers						
18–24					46.2 %	27.8 %
25–44					67.0 %	47.2 %
45–64					34.9 %	27.0 %
65 and over					17.6 %	17.3 %
Panel 4: individuals 18 and over who engaged in any interactive child care and interactive adult care						
Age of caregivers						
18–24					5.7 %	2.5 %
25–44					7.8 %	4.8 %
45–64					3.8 %	2.6 %
65 and over					1.0 %	1.0 %
Panel 5: individuals 18 and over who engaged in any interactive child care and interactive adult care *except* for “helping a non-household adult”						
Age of caregivers						
18–24					2.1 %	0.8 %
25–44					3.3 %	2.2 %
45–64					1.5 %	1.3 %
65 and over					0.3 %	0.4 %
Panel 6: Individuals 18 and over who engaged in any child care (interactive or supervisory) or interactive adult care						
Age of caregivers						
18–24					54.7 %	35.9 %
25–44					77.2 %	62.5 %
45–64					40.9 %	34.6 %
65 and over					19.7 %	19.5 %
Panel 7: individuals 18 and over who engaged in any child care (interactive or supervisory) and interactive adult care						
Age of caregivers						
18–24					7.2 %	3.8 %
25–44					8.9 %	6.5 %
45–64					4.7 %	3.6 %
65 and over					1.3 %	1.3 %

Panel 2 shows that participation in any interactive adult care (broadly defined to include helping a non-household adult) followed a very different pattern. Women in every age group except 65 and over were more likely to engage in child care than adult care. But men in two age groups—the 18–24 category as well as the 65 and over category—were more likely to provide adult than child care. Further, the gender differences in adult care were much smaller than those in child care. In both the youngest and the oldest age group men were more likely than women to provide care for an adult. Further, men in the 25–44 and 45–64 age groups were almost as likely as women to provide adult care.

Panel 3 shows the percentage of women and men who engaged *either* in child care or adult care on the diary day. Note that these percentages do not equal the sum of the percentage engaging in any child care and any adult care, because of overlaps. That is, a number of women and men engaged in both types of care activity on the diary day. These results are interesting primarily because they show that a relatively high percentage of men as well as women provided at least one form of care on a given day, ranging from a low of 17.3 % for men 65 and over to a high of 67 % for women ages 25–44.

Panels 1–3 set the context for the focus on sandwich caregivers in later panels by distinguishing them from non-sandwich givers who engaged only in child care or adult care. Panel 4 offers a definition of sandwich caregivers based only on interactive care, tallying the percentage of individuals who engaged in both child care and adult care on a diary day. By this definition, the overall share of sandwich caregivers appeared relatively small, ranging from a high of 7.8 % among women aged 25–44 to lows of 1 % for both women and men 65 and over. Among men aged 25–55 almost 5 % (4.8 %) could be characterized as sandwich caregivers on a given day. However, as Panel 5 demonstrates, the percentages were much lower when “helping a non-household adult” was excluded from the definition. A noticeable reduction was apparent for every gender/age group.

Panels 1–5 all ignore supervisory care for children under the age of 13. Inclusion of this form of supervisory care has a huge impact on the percentage engaged in either child or adult care, as can be seen from a comparison of Panel 6 with Panel 3.^7^ The percentage of women providing some form of care (when supervisory care is included) exceeded 50 % for two age groups, and was remarkably high (at 77.2 %) for women aged 25–44. The percentage of men aged 25–44 providing at least one form of care was also very high, at 62.5 %.

Panel 7 shows the implications of including supervisory care for children in a definition of sandwich care. The percentages caring for both a child and an adult on the diary day were greater for every gender/age category, compared to Panel 4. They were highest for women aged 25–44, at 8.9 %; second highest for women aged 18–24, at 7.2 %; and third highest for men aged 25–44, at 6.5 %.

### Temporal Burden of Care

Assessing the relative temporal burden of sandwich care required an analysis of differences in the average amount of time devoted to care for both children and adults. Table 3 shows differences in means among those who provided at least some kinds of care on a diary day, and those who provided sandwich care. Regardless of how sandwich care is defined, it involves greater temporal burdens than simply providing at least one form of care. Panel 2 compares sandwich caregivers, defined only in terms of interactive child care and adult care, with those who provided at least some care. Not surprisingly, women ages 45–64 devoted more time on average to this form of sandwich care—3.3 h. Yet there is less variation across gender and age in Panel 2 than in Panel 1, suggesting that when “dual responsibilities” were incurred, their temporal demands were great regardless of the caregivers’ demographic characteristics.

**Table 3:** Mean daily hours devoted to interactive child care (and supervisory care) and adult care, by gender and age of sandwich caregivers (ATUS 2003–2012, hours per day)

	Women	Men
	Mean hours per day provided by those engaged in activity	Mean hours per day provided by those engaged in activity
Panel 1: individuals 18 and over who engaged in any interactive child care or interactive adult care		
Age of caregivers		
18–24	2.1	1.5
25–44	2.6	1.9
45–64	1.9	1.7
65 and over	1.7	1.8
Panel 2: individuals 18 and over who engaged in any interactive child care and interactive adult care		
Age of caregivers		
18–24	3.2	2.5
25–44	3.3	2.7
45–64	2.8	2.8
65 and over	2.9	2.7
Panel 3: individuals 18 and over who engaged in any interactive child care and interactive adult care *except* for “helping a non-household adult”		
Age of caregivers		
18–24	3.3	2.3
25–44	3.2	2.6
45–64	2.6	2.6
65 and over	2.8	2.1
Panel 4: individuals 18 and over who engaged in any child care (interactive or supervisory) or interactive adult care		
Age of caregivers		
18–24	7.1	3.7
25–44	9.7	6.7
45–64	5.4	4.7
65 and over	3.5	3.0
Panel 5: individuals 18 and over who engaged in any child care (interactive or supervisory) and interactive adult care		
Age of caregivers		
18–24	8.8	6.5
25–44	10.9	8.2
45–64	7.1	6.7
65 and over	5.7	4.9

Panel 3 shows the consequences of excluding the activity category “helping a non-household adult” compared to Panel 2. As noted earlier, “helping a non-household adult” includes support work such as cleaning and cooking. Therefore, exclusion of this category made adult care estimates more comparable to child care estimates. Interestingly, this exclusion, which had a noticeable impact on the percentage engaged (Table 2) had only small implications for the mean amount of time devoted to sandwich care. While the difference for men 65 and over amounted to 0.6 h, the difference for other age/gender categories was never greater than 0.2 h. Panels 4 and 5 extend the comparison to include supervisory care for children, comparing those who provided care for both. As with the comparison between Panels 1 and 2, it is apparent that sandwich caregivers devoted far more time to care. For women in the age category 25–44, the average time reached 10.9 h per day, compared to 9.7 h for those who engaged in one or the other. By this definition, sandwich caregivers truly shoulder a significant burden. Even the sandwich caregiver group with the lowest mean, men aged 65 and over, spent 4.9 h per day, on average, in care provision. It is important to note, as aforementioned, that this estimate did not include supervisory care for adults, which could represent a significant responsibility for both women and men in this age group engaged in spousal care.

Assessment of the relative burden of child care and adult care for sandwich caregivers required disaggregation by the age of care recipient. Table 4 shows the relative burden of child care and adult care across differently defined sandwich caregivers, following the same sequence as Table 3 (note that Panels 1 and 4 include overlaps between child care and adult care, so the percentage engaged in both is not equal to the sum of the percentage engaged in either). Panel 2 in Table 4 represents what might be termed a “conventional” definition of sandwich caregiving: those who engaged in both interactive child care and adult care. For both women and men, both the frequency of engaging in child care and the average amount of time devoted to child care exceeded the corresponding estimates for adult care. This was true even for men and women aged 65 and over, perhaps attesting to the important role of grandparental responsibilities.

**Table 4:** Disaggregation of total unpaid care into child care and adult care by gender and age of unpaid caregivers (ATUS 2003–2012, hours per day)

	Interactive child care				Interactive adult care				Supervisory care			
	Women		Men		Women		Men		Women		Men	
	Engaged in activity on diary day	Mean hours per day provided by those engaged in activity	Engaged in activity on diary day	Mean hours per day provided by those engaged in activity	Engaged in activity on diary day	Mean hours per day provided by those engaged in activity	Engaged in activity on diary day	Mean hours per day provided by those engaged in activity	Engaged in activity on diary day	Mean hours per day provided by those engaged in activity	Engaged in activity on diary day	Mean hours per day provided by those engaged in activity
Panel 1: individuals 18 and over who engaged in any interactive child care or interactive adult care^a^												
Age of caregivers												
18–24	69.1 %	1.6	37.7 %	0.7	43.1 %	0.5	70.3 %	0.8				
25–44	91.8 %	2.3	81.3 %	1.5	20.1 %	0.2	29.0 %	0.4				
45–64	61.7 %	1.2	58.8 %	1.0	49.4 %	0.7	49.9 %	0.8				
65 and over	37.8 %	0.7	31.4 %	0.6	68.4 %	1.0	74.7 %	1.2				
Panel 2: individuals 18 and over who engaged in any interactive child care and interactive adult care^b^												
Age of caregivers												
18–24	64.0 %	2.0	60.5 %	1.5	36.0 %	1.1	39.5 %	1.0				
25–44	68.7 %	2.3	59.4 %	1.6	31.3 %	1.0	40.6 %	1.1				
45–64	61.9 %	1.7	56.6 %	1.6	38.1 %	1.1	43.4 %	1.2				
65 and over	59.5 %	1.7	56.3 %	1.5	40.5 %	1.2	43.7 %	1.2				
Panel 3: individuals 18 and over who engaged in any interactive child care and interactive adult care *except* for “helping a non-household adult”^c^												
Age of caregivers												
18–24	72.0 %	2.4	62.2 %	1.4	28.0 %	0.9	37.8 %	0.9				
25–44	76.2 %	2.4	67.3 %	1.7	23.8 %	0.8	32.7 %	0.8				
45–64	63.6 %	1.7	64.5 %	1.7	36.3 %	0.9	35.5 %	0.9				
65 and over	57.7 %	1.6	60.5 %	1.3	42.3 %	1.2	39.5 %	0.8				
Panel 4: individuals 18 and over who engaged in any child care (interactive or supervisory) or interactive adult care												
Age of caregivers												
18–24	21.1 %	1.4	15.2 %	0.5	6.1 %	0.4	18.1 %	0.6	72.8 %	4.8	66.7 %	2.3
25–44	23.0 %	2.0	17.6 %	1.1	2.3 %	0.2	5.0 %	0.3	74.7 %	6.7	77.4 %	5.0
45–64	21.6 %	1.0	17.1 %	0.8	13.3 %	0.6	14.1 %	0.6	65.1 %	3.1	68.8 %	3.1
65 and over	22.4 %	0.7	19.3 %	0.5	29.6 %	0.9	37.0 %	1.0	48.0 %	1.4	43.8 %	1.2
Panel 5: individuals 18 and over who engaged in any child care (interactive or supervisory) and interactive adult care												
Age of caregivers												
18–24	19.2 %	1.6	15.8 %	1.0	13.6 %	1.1	16.8 %	1.0	67.2 %	5.6	67.4 %	4.2
25–44	20.3 %	2.1	15.7 %	1.2	10.6 %	1.1	16.3 %	1.3	69.0 %	7.0	68.1 %	5.4
45–64	21.8 %	1.4	18.0 %	1.2	18.4 %	1.2	21.2 %	1.4	59.8 %	3.8	60.8 %	3.9
65 and over	26.6 %	1.4	27.1 %	1.2	22.2 %	1.2	26.9 %	1.2	51.3 %	2.7	46.0 %	2.1

^a,b,c^Supervisory care in panels 1–3 is excluded, which results in blank cells

Also noteworthy are the gender differences among sandwich caregivers as defined in Panel 2. Conditional on fitting these sandwich criteria, men and women were more similar in both probability and level of engagement. Consistent with earlier discussion, men who are sandwich caregivers were more likely than women to be providing adult care, and mean levels of care provided were quite similar. As Panel 3 indicates, exclusion of the category “helping a non-household adult” tilted both the percentages and levels more toward child care, and away from adult care. In other words, this exclusion has very important implications for the assessment of the relative burden of children and adults on sandwich caregivers. Since doing household chores or helping with instrumental activities of daily living for children (i.e., support care for children) are generally not considered child care, this exclusion improved consistency of comparison between care for the two age groups.

Panels 4 and 5 demonstrate the effect of including supervisory child care on the relative burden of children and adults among sandwich caregivers defined in these terms. An inclusion of supervisory care further tilted the distribution of care among sandwich caregivers toward children. More than 50 % of those in most of the age/gender categories engaged in supervisory care. A noticeable difference between Panels 4 and 5 is that men and women doubled their time on adult care even though fewer are engaged in adult care activities.

In order to summarize the challenges posed by definitional differences, Table 5 provides lower-, middle-, and upper-bound estimates of participation in child care and adult care and the relative burden imposed by child care and adult care. The lower bound was defined by participation in interactive child care and interactive adult care excluding “helping a non-household adult” activities. The middle bound dropped this exclusion. As can be seen from Table 5, this had a large effect on the percentage of both women and men (in every age group) who are defined as sandwich caregivers, but had only a small effect on the means. The upper bound included supervisory care as a criterion for participation, and added both supervisory care and an estimate of support care within the household to the estimates of mean hours. Participation rates were uniformly higher for all age and gender groups. Most striking, however, is the increase in mean hours per day, which reached 10.9 for women aged 25–44 and 8.2 for men aged 25–44.

**Table 5:** Lower bound, middle bound, and upper bound of sandwich caregivers’ responsibilities, by gender and age of sandwich caregivers (ATUS 2003–2012, hours per day)

	Women		Men	
	Engaged in activity on diary day	Mean hours per day provided by those engaged in activity	Engaged in activity on diary day	Mean hours per day provided by those engaged in activity
Lower bound (individuals 18 and over who engaged in any interactive child care and interactive adult care excluding for “helping a non-household adult”)^a^				
Age of caregivers				
18–24	2.1 %	3.3	0.8 %	2.3
25–44	3.3 %	3.2	2.2 %	2.6
45–64	1.5 %	2.6	1.3 %	2.6
65 and over	0.3 %	2.8	0.4 %	2.1
Middle bound (individuals 18 and over who engaged in any interactive child care and interactive adult care)^b^				
Age of caregivers				
18–24	5.7 %	3.2	2.5 %	2.5
25–44	7.8 %	3.3	4.8 %	2.7
45–64	3.8 %	2.8	2.6 %	2.8
65 and over	1.0 %	2.9	1.0 %	2.7
Upper bound (individuals 18 and over who engaged in any child care (interactive and supervisory) and interactive adult care)^c^				
Age of caregivers				
18–24	7.2 %	8.8	3.8 %	6.5
25–44	8.9 %	10.9	6.5 %	8.2
45–64	4.7 %	7.1	3.6 %	6.7
65 and over	1.3 %	5.7	1.3 %	4.9

^a^Lower bound is calculated by total hours spent on interactive child care and interactive adult care subtracting the time spent for helping a non-household adult. Housework hours are calculated by total housework hours subtracting an approximation of housework done for “self” (subtracting per capita housework hours)

^b^Middle bound is calculated by total hours spent on interactive child care and interactive adult care

^c^Upper bound is calculated by total hours spent on any child care including interactive child care and supervisory child care and interactive adult care and support care for others

In sum, if “care” is defined narrowly as engagement in interactive child care and adult care, relatively few adults—less than 3.5 %, even for those in the prime caregiver group of women aged 25–44—provided care and they devoted between 2.1 and 3.3 h to these activities on a diary day. If “care” is defined broadly to include supervisory responsibilities and support care (if provided by those engaged in interactive or supervisory child care and interactive adult care), both participation rates and means were far higher. The percentage of those aged 25–44 who could be described as sandwich caregivers on the diary day rose to 8.9 % for women and 6.5 % for men. The mean hours for every group except for those 65 and over amounted to more than 6 h per day. This implies a weekly average of more than 40 h a week, that is, more than a full-time job, as conventionally defined.

### Crosswalk with Different Surveys

Ideally, estimates of the average daily burden of sandwich care would be combined with estimates of the distribution of care episodes over time. As aforementioned, adult care in particular is likely to be distributed less evenly throughout the year than child care. To address this, daily time-use surveys (ATUS) can be complemented by surveys focusing on care episodes. In order to increase comparability between time-diary estimates and estimates based on surveys of assistance with ADLs and IADLs, I used the crosswalk between these two approaches described earlier to estimate time in specific IADLs (ADLs cannot be disaggregated from ATUS codes) in Table 6.

**Table 6:** Daily time devoted to ADLs and IADLs by gender of caregivers and type of care recipient (ATUS 2003–2012, minutes per day, for those who provided some ADLs and IADLs for children and adults)

	Children		Adults	
	Women	Men	Women	Men
Lower bound (individuals 18 and over who engaged in any interactive child care and interactive adult care excluding for “helping a non-household adult”)				
ADLs	49	25	11	4
IADLs	143	105	103	83
Housework/laundry^a^	25	8	24	11
Meal preparation^b^	24	12	23	10
Shopping^c^	13	11	13	11
Travel	21	22	21	26
Management^d^	11	11	11	16
Getting around outside	5	5	3	4
Taking medication^e^	3	2	8	7
Developmental care (for children)^f^	41	34	n.a.	n.a.
Total of ADLs and IADLs	192	130	114	87
Middle bound (individuals 18 and over who engaged in any interactive child care and interactive adult care)				
ADLs	44	23	6	2
IADLs	140	99	93	71
Housework/laundry^a^	23	7	19	8
Meal preparation^b^	21	10	18	8
Shopping^c^	16	10	15	9
Travel	20	20	27	30
Management^d^	11	10	8	10
Getting around outside	7	6	2	3
Taking medication^e^	3	2	5	4
Developmental care (for children)^f^	39	34	n.a.	n.a.
Total of ADLs and IADLs	184	122	99	74
Upper bound (individuals 18 and over who engaged in any child care (interactive and supervisory) and interactive adult care)				
ADLs	38	17	7	2
IADLs	125	80	96	74
Housework/laundry^a^	21	6	19	7
Meal preparation^b^	19	9	18	7
Shopping^c^	15	10	16	10
Travel	17	15	28	32
Management^d^	9	7	9	11
Getting around outside	7	5	2	3
Taking medication^e^	3	2	5	3
Developmental care (for children)^f^	33	26	n.a.	n.a.
Total of ADLs and IADLs	163	97	103	76

^a,b,c^Activities in IADLs are calculated by those activities done for adults and children separately (calculated per capita and multiplied by the number of household adults (except for self) for IADLs for adults and calculated per capita and multiplied by the number of household children for IADLs for children)

^d^Management for adults is specific to financial management, while management for children includes activities like managing events for children

^e^Taking medication for children are calculated by care activities related to children’s health

^f^Developmental care is specific to children

For activities like housework, including doing laundry and meal preparation as IADL categories, there are no questions asking “who for” in the ATUS. In order to adjust for this weakness and estimate the amount of IADL work that can be attributed to children or adults needing assistance, I divided the average time devoted to housework, meal preparation, and shopping by the number of total household members (per capita hours). I then multiplied those by the number of children in the household and by the number of household adults (except for oneself) in the household. Both women and men spent more time devoted to ADLs and IADLs for children than for adults. In general, men made larger relative contributions to IADL time than to ADL time. Overall, the results in Table 6 suggest that IADLs were far more time-consuming than ADLs, though this may partly reflect lack of disaggregation in the activity codes. It is worth noting, again, that the supervisory demands of adult care are not explicitly included in either measure.

Finally, I used this method of assigning average time use to compare time devoted to ADLs and IADLs as measured by the ATUS with those provided by stylized surveys (Table 7). Weekly hours devoted to ADLs and IADLs were calculated by daily hours of ADLs and IADLs multiplied by 7. The 1994 Health and Retirement Survey (HRS) indicated that among active caregivers (those who provide at least some hours of elderly care) the average number of care hours per week was 19.4 (Amirkhanyan and Wolf 2003). The 1996 Survey of Income and Program Participation (SIPP) reported that those who provide unpaid care or assistance to someone with long-term illness or disability during the past month spent on average 24.2 h per week (Alecxih et al. 2001). The 1997/1999 National Longitudinal Survey of Young Women (NLSYW) reported that the female sandwich-caregivers between the ages of 45 and 54 who spend at least some time to care for children and parents devoted 49.2 h per week to unpaid care. One reason for this comparatively high estimate by NLSYW may be that the caregivers were limited to females in prime sandwich caregiving ages.

**Table 7:** Crosswalk of weekly hours of unpaid care

Data sources	Definition of caregivers	Average caregiving hours
1994 Health and Retirement Study (Amirkhanyan and Wolf 2003)	Among those who spent 100 or more hours in the past 12 months helping parent(s) (or stepparents) with basic personal needs like dressing, eating, and bathing excluding time spent on transport, shopping, cooking, and paying bills	19.4
2003–2012 American time use survey^a^	Among those who are 18 and over and spend at least 1.2 h on a diary day engaging in any interactive adult care excluding the time on transport, shopping, cooking, and paying bills^1^	21.4
2002 Health and Retirement Study (Johnson and Schaner 2005)	Among those 54–64 who provided at least 100 h of care for grandchildren and parent/spouse care in the previous two years	11.2
2003–2012 American time use survey	Among those 54–65 who provide at least some care for children and adults on a survey day	27.6
1996 survey of income and program participation (Alecxih et al. 2001)	Among those who provide unpaid care or assistance to someone with a long-term illness or disability during the past month	24.2
2003–2012 American time use survey	Among those 18 and over who provide some adult care	14.0
1997/1999 National longitudinal survey of young women (Pierret 2006)	Among women age 45 and 54 who provide some time for children and parents	49.2
2003–2012 American time use survey	Among women age 45 and 54 who provide some time for children and adults	37.0
2009 National alliance for caregiving/AARP	Among those 18 and over who provide unpaid care to a relative or friend (18+) or any child (<18) in the last 12 months	18.8
2003–2012 American time use survey	Among those 18 and over who provide unpaid care to non-household adults or any child (<18) on a survey day	21.8
2003–2012 American time use survey lower bound	Among individuals 18 and over who engaged in any interactive child care and interactive adult care excluding for “helping a non-household adult”)	20.0
2003–2012 American time use survey middle bound	Among individuals 18 and over who engaged in any interactive child care and interactive adult care)	20.9
2003–2012 american time use survey upper bound	Among individuals 18 and over who engaged in any child care (interactive and supervisory) and interactive adult care)	60.0

^a^Estimates provided by 2003–2012 American time use survey data are weekly average hours converted by multiplying daily hours by 7

The 2002 HRS (Johnson and Schaner 2005) exhibited the lowest estimate of average caregiving hours to grandchildren and parents/spouse (11.2 h per week) because caregivers report the number of hours they had spent on caregiving over a two-year period rather than estimate the hours spent in a “typical” or “usual” week.^8^ The 2009 NAC/AARP, on the other hand, estimated typical hours spent on caregiving for those who are aged 18 and over and spent some time on care for any child (<18) and relative or friend (18+) in the last 12 months.

For comparison with these surveys, I generated estimates of time devoted to care from the ATUS that are as consistent as possible with these sources. Differences in the time period covered also limited comparability. In some cases, notably comparisons with the 1994 Health and Retirement Study and the 2009 National Alliance for Caregiving survey, the estimates from these very disparate sources were quite similar. In other cases, such as the 2002 Health and Retirement Survey and the 1996 Survey of Income and Program Participation, they varied by more than 100 %. The empirical challenges, while significant, are perhaps less disconcerting than conceptual and measurement ones. The use of a time-use survey (ATUS) offers a helpful perspective on ways in which data-collection efforts can be modified and expanded. Nonetheless, as shown previously, existing time-use surveys also suffer from a number of serious limitations, including issues related to supervisory care and support care (especially for non-household adults). Further, many time-use surveys, including ATUS, collect data on only one person per household for 1 day, making it difficult to estimate the total care burden for sandwich caregivers. Clearly, further efforts to improve consistency of estimates within surveys and across them are required.

## Conclusion

The need for accurate measures of sandwich care responsibilities grows out of practical concerns as well as research priorities. Improved measures could help assess the costs of sandwich care, which often include a significant reduction in market income, especially for women. Such assessments are relevant both to public policies providing support to family caregivers and to private insurance markets for long-term care.

This paper offers three important contributions to measurement of the temporal burden carried by sandwich caregivers. First, it highlights important conceptual and definitional problems that have often been compounded by inconsistency in survey designs. Second, it shows that, despite these problems, analysis of the American time use survey (ATUS) provides useful comparisons of the temporal burden of combined child care and adult care and the distribution of this burden between women and men. Third, it shows how data from the ATUS can be used to both compare and calibrate results from stylized surveys based on questions regarding assistance with activities of daily living and instrumental activities of daily living.

However, the results presented here are limited in several aspects. While more detailed measures of care work provide important insights, they also generate more complex results. No single number adequately captures the burden of sandwich care, putting more responsibility on those interpreting the results presented here to choose which measures are more relevant to specific policy concerns. While crosswalk analysis is useful, it remains approximate. As is often the case, more detailed analysis of existing data reveals the need for improved survey design.

It is sometimes said that “you can’t manage what you don’t measure.” While this may not be literally true, it will be difficult to assess the impact of economic and demographic change on family caregiving without a clear picture of its quantitative dimensions. This paper generates two important recommendations for survey design. First, researchers should try to move toward more consistent and detailed definitions of care provision, whether provided on behalf of children or needy adults. Second, researchers should encourage efforts to develop larger, more unified surveys that could take the place of many small surveys, combining time-diary and stylized data. Ideally, qualitative research could also be designed to help assess and calibrate quantitative measures.

## Footnotes

1 I use the term “adult” rather than “elderly person” or “frail elderly” because the ATUS does not distinguish between care for different types of adults.

2 One exception is the special module on elder care (2011–2014 elder care questionnaire in ATUS). The questionnaire asks more detailed questions about elder care recipients including the age of elder care recipient, the relationship to caregiver, the type of residence (household vs. non-household), and duration of care for the elderly.

3 More details from this table available at http://www.bls.gov/news.release/atus.t10.htm.

4 Among its categories of primary adult care, the ATUS data includes “looking after adults.” However, it is recognized as a primary activity, rather than as a “supervisory activity,” as described in child care.

5 Prior to time-use surveys, which employ the diary-based method of reporting, many estimates of care work (and non-market work in general), were based on respondents’ answers to “stylized” questions about amounts of time spent on an average day or an average week. For instance, the Panel Study of Income Dynamics (PSID) asks “[a]bout how much time do you spend on housework in an average week—I mean time spent cooking, cleaning, and doing other work around the house?”.

6 The American Time Use Survey User’s Guide by Bureau of Labor Statistics (2014) provides more details on sampling and weighting procedures.

7 For a more detailed discussion of the importance of inclusion of supervisory care in measuring child care and the related economic consequences, see Suh and Folbre (2015).

8 SIPP and NAC/AARP studies estimated the hours spent in a “typical” or “average” week.
